# Dendritic cells provide a potential link between smoking and inflammation in rheumatoid arthritis

**DOI:** 10.1186/ar4046

**Published:** 2012-10-04

**Authors:** Marina G Kazantseva, John Highton, Lisa K Stamp, Paul A Hessian

**Affiliations:** 1 Department of Physiology, University of Otago, P.O. Box 913, Dunedin 9054, NZ; 2 Department of Medicine, University of Otago, P.O. Box 913, Dunedin 9054, NZ; 3 Department of Medicine, University of Otago, P.O. Box 4345, Christchurch 8041, NZ

## Abstract

**Introduction:**

Smoking increases the risk of developing rheumatoid arthritis (RA) and affects the severity of established RA. Smoking can impact on Th17 lymphocyte differentiation and function through activation of the aryl hydrocarbon receptor (AHR), a process with implications for the pathogenic mechanisms in RA that involve the cytokine, interleukin (IL)-17A. The objective of this study was to establish any effect of smoking on the inflammatory tissue lesions of rheumatoid arthritis via the AHR and IL-17A.

**Methods:**

Twenty synovial and eighteen subcutaneous nodule tissue samples from 31 patients with RA were studied. Patient smoking status at the time of tissue collection was established. Expression of *AHR*, *CYP1A1*, *AHRR*, *IL6*, *IL17A*, *IL17F*, *IL22*, *IL23*, *IL23R*, *IFNG*, *TBX21*, *IDO1 *and *FOXP3 *genes were assessed in tissues and cultured cells using real-time PCR. Two-colour immunofluorescence was used to co-localise AHR and CYP1A1 protein in synovial tissues. The response of monocytes and monocyte-derived dendritic cells (mo-DCs) to the AHR agonist, benzo(*a*)pyrene (B*a*P) was compared *in vitro*.

**Results:**

*AHR *gene expression was demonstrated in rheumatoid synovial tissues and nodules with significantly greater expression in synovia. Expression was not influenced by smoking in either tissue. Evidence of AHR activation, indicated by *CYP1A1 *and *AHRR *gene expression, was found only in synovia from patients who smoked. However, *IL17A *gene expression was lower in synovia from smokers. *TBX21 *and *FOXP3 *expression was not affected by smoking. Within the synovial tissues of smokers the principal cell type with evidence of AHR activation was a subset of synovial DCs. This observation was consistent with the sensitivity of human mo-DCs to B*a*P stimulation demonstrated *in vitro*. Exposure to B*a*P affected mo-DC function as demonstrated by decreased *IL6 *expression induced by PolyI:C, without affecting indoleamine 2,3 dioxygenase (IDO)1 expression.

**Conclusion:**

Our findings show that one effect of smoking on inflamed rheumatoid synovial tissue involves activation of the AHR pathway. A subset of synovial DCs is important in the response to cigarette smoke. The potential for smoking to affect DC behaviour in joint tissues has relevance to both early and late phases of RA pathogenesis and warrants further investigation.

## Introduction

Rheumatoid arthritis (RA) is a systemic autoimmune disease predominantly manifest as polyarthritis but with extra-articular complications such as rheumatoid nodules (granulomas) in more severe cases. Clinical evidence points to an effect of smoking on the severity of established RA. Patients with RA who continue to smoke cigarettes have higher disease activity and develop worse disability [1,2]. They have a greater requirement for treatment with disease-modifying antirheumatic drugs (DMARDs) [3] and respond less well to anti-TNF agents [4,5]. Smokers with RA are also less likely to achieve sustained DMARD-free remission than non-smokers [6].

Interactions between genetic pre-disposition and environmental factors have been identified as important in determining the risk of developing RA. Approximately 50% of the risk is attributable to genetic factors with HLA-DRB1 shared epitope (SE) alleles the major genetic determinants of RA susceptibility [7,8] and severity [9,10]. Other genetic risk loci particularly associated with the development of anti-citrullinated peptide antibody (ACPA)-positive RA, include genes that influence T cell function and the handling of arthritogenic antigens [11-13].

Epidemiologic data has established cigarette smoking as an important environmental factor that interacts powerfully with the SE to increase the risk for development of RA [14-16]. Smoking is associated with increased production of autoantibodies, including ACPA and rheumatoid factor (RF) and with increased incidence of extra-articular manifestations in RA that include the development of rheumatoid nodules [16,17]. Biologic mechanisms that explain the epidemiologic data and accommodate an influence of the SE are increasingly understood [15,18,19]. One aspect is that smoking enhances the expression of peptidylarginine deiminase and consequently increases the generation of citrullinated protein(s) within the lung alveolar compartment [20]. There is evidence that antibodies reacting with citrullinated whole proteins, contribute to the pathogenesis of RA. These include antibodies to citrullinated fibrinogen or collagen type II that are involved in immune-complex mediated inflammation as well as antibodies to citrullinated α-enolase, which are particularly associated with SE^+ ^*HLA-DRB1 *alleles and that identify patients with a higher frequency of joint erosions and RF positivity [21-23]. Furthermore, T cells in RA patients also respond to citrullinated aggrecan peptides [24]. Thus, smoking and interactions between smoking and genetic variants contribute to autoimmunity against post-translationally modified (citrullinated) peptides/proteins that are important in the pathogenesis of RA [25].

Of further relevance is the potential for smoking to influence T helper (Th)17 lymphocyte-mediated inflammation. Polycyclic aromatic hydrocarbons (PAHs) are amongst a number of compounds present in cigarette smoke that activate the aryl hydrocarbon receptor (AHR), a transcription factor that binds to xenobiotic response elements (XRE) and regulates gene expression. Genes encoding select members of the cytochrome P450 (CYP) family of enzymes, (for example, *CYP1A1*), and the AHR repressor (*AHRR*) are particularly responsive to ligand-dependent AHR activation, a characteristic used to distinguish AHR activation. *In vitro *evidence shows that the activation of AHR also promotes the differentiation of Th17 lymphocytes and consequently the production of the Th17-related cytokines, interleukin (IL)-17A, IL-17F and IL-22 [26]. Experimental models of arthritis and clinical indications have highlighted an important role for IL-17A in the pathogenesis of RA [27-29]. Thus the AHR provides another potential link between exposure to compounds in cigarette smoke and the notable effect that smoking has on rheumatoid inflammation. To address this possibility, we set out to establish the presence of the AHR in the tissues of patients with RA, to seek evidence for activation of the AHR pathway in joint and extra-articular sites of inflammation in smokers and non-smokers, and to investigate corresponding levels of *IL17A *expression. Our results indicate AHR activation in synovial tissue, associated with smoking. Synovial dendritic cells are sensitive to AHR ligand and in RA patients respond with activation of the AHR. Contrary to expectation the activation of AHR in synovial tissue was not associated with increased IL-17A expression.

## Materials and methods

### Patients and tissue samples

Twenty synovial and eighteen nodule tissue samples were obtained from 31 patients with RA. All patients fulfilled American Rheumatism Association 1987 classification criteria for RA [30]. Patients were classified according to their reported smoking habits as either: (i) smokers (actively smoking when the tissue sample was taken) - including those who were daily or non-daily smokers of tobacco via cigarettes or loose tobacco; or (ii) non-smokers - including ex-smokers (those who ceased smoking ≥ 3 months prior to nodule samples or ≥ 8 years prior to synovial samples being taken), and those who have never smoked tobacco in their lifetime. The demographics of RA patients contributing synovial and/or nodule tissue are summarised in Table 1. With two exceptions, all RA patients included in this study were SE^+ ^(overall single copy of SE, n = 20 (65%); double copy of SE, n = 9 (29%)).

**Table 1:** Patient demographics and clinical data

	Synovia		Nodules	
	Smokers(n = 6*)	Non-smokers(n = 11*)	Smokers(n = 4)	Non-smokers(n = 14)
Female, %	83	64	100	71
Age, years**	58 (37-79)	55 (35-74)	47 (44-50)	61(48-77)
Disease duration, years**	11 (2-30)	18 (3-30)	19 (13-30)	19 (2-44)
Nodules, n	5 (83%)	9 (82%)	4 (100%)	14 (100%)
Erosions, n	6 (100%)	10 (91%)	4 (100%)	13 (93%)
RF positive, n	6 (100%)	10 (91%)	4 (100%)	13 (93%)
ACPA Positive^†^, n/total ACPA Titer^††^	5/5 (100%) 157 ± 45	8/9 (89%) 177 ± 26	4/4 (100%) 193 ± 57	12/13 (92%) 157 ± 22
ESR**	34 (3-85)	33 (13-64)	30 (13-64)	33 (6-54)

* Three patients (one smoker, two non-smokers) provided two synovial samples. ** mean values with the range in parenthesis. ^†^For some patients, tissue samples pre-dated the availability of tests for anticitrullinated peptide antibody (ACPA); ^†† ^Mean values ± SEM. Mean ACPA titers were not significantly different. n, number of patients; RF, rheumatoid factor; SEM, standard error of the mean,]; ESR, erythrocyte sedimentation rate.

Two synovial samples were available from three patients (one smoker, two non-smokers), taken 6 months, 1 year and 5 years apart respectively. Paired synovial and nodule samples were obtained at the same time from four separate patients (one smoker, three non-smokers).

Synovia from two patients with osteoarthritis (OA) were also studied. One synovium (male patient, age 66 years) was processed commercially (Origene, Rockville, Maryland, USA); the second synovium (female patient, age 72 years) was sourced from tissue stocks. Their smoking status is unknown.

Informed consent was obtained for the use of all patient tissue, with the study approved by the New Zealand Multi-Regional Ethics Committee. The use of peripheral blood (PB) from normal healthy donors and associated work involving PB leukocytes was approved by the University of Otago Ethics committee.

### Isolation, culture, and stimulation of PB monocytes and monocyte-derived dendritic cells (mo-DCs)

Peripheral blood mononuclear cells were isolated from heparinized venous blood of healthy non-smoking human donors by Ficoll density centrifugation. CD3^+ ^T-cells, CD20^+ ^B-cells, and CD14^+ ^monocytes were purified using appropriate antibody-coated magnetic MACS^® ^microbeads and an AutoMacs separator (Miltenyi Biotec, Bergisch Gladbach, Germany) according to the manufacturer's instructions. Lymphocytes were used without further manipulation.

CD14^+ ^monocytes were resuspended in RPMI 1640 medium supplemented with 10% fetal calf serum, 2mM glutamine, 50 U/ml penicillin, 50 μg/ml streptomycin (complete medium) and were stimulated with varied concentrations (0.001μM to 10 μM) of benzo(*a*)pyrene (B*a*P) or equivalent dimethyl sulfoxide (DMSO) alone (control) for 24 hr. Stimulation with B*a*P, a polycyclic aromatic hydrocarbon present in cigarette smoke mimicked the effect of smoke. Alternatively, mononcytes were stimulated with 10μM phorbol 12-myristate 13-acetate (PMA) in complete medium for 72 hr at 37ºC and 5% CO_2_.

Mo-DCs were generated from CD14^+ ^monocytes by culture in complete medium with recombinant human granulocyte macrophage colony-stimulating factor (rhGMCSF), and rhIL-4 (each 25ng/ml; R&D Systems, Minneapolis, Minnesota, USA) for 6 days at 37ºC and 5% CO_2_. Complete medium containing cytokines was replenished on day 3. Mo-DCs were further treated with either DMSO or 0.001μM to- 10 μM B*a*P in DMSO, or with polyinosinic:polycytidylic acid (Poly I:C) (12.5μg/ml) for 24 hr. Purity and maturation of DCs was assessed by staining with monoclonal antibodies to CD14, CD11c and CD83 (BD Biosciences, San Jose, California, USA) and FACS analysis. Immature mo-DCs were CD14^-^, CD11c^+^, CD83^low ^and as expected became CD14^-^, CD11c^+^, CD83^high ^upon maturation induced by Poly I:C stimulation.

### Quantitation by real-time polymerase chain reaction (RT-PCR)

Total RNA was purified from rheumatoid nodule and synovial tissues or from cultured cells, and reverse transcribed using Superscript III (Invitrogen, Carlsbad, California, USA) as previously described [31]. Gene expression was assessed by RT-PCR using SYBR Green or TaqMan gene expression assays on an Applied Biosystems ABI 7300 sequence detection system. The SYBR Green primers (Invitrogen) were *AHR *(sense): 5'-TTC AGT TCT TAG GCT CAG CGT -3', *AHR *(antisense): 5'-TGC TGC TCT ACA GTT ATC CTG G -3' and *GAPDH *(sense): 5'- TGC ACC ACC AAC TGC TTA GC -3', *GAPDH *(antisense): 5'- GGC ATG GAC TGT GGT CAT GAG -3'. Commercially available Taqman assays (Applied Biosystems, Foster City, California, USA) were used to measure *CYP1A1 *(Hs00153120_m1), *AHRR *(Hs01005075_m1), *IL17A *(Hs00174383_m1), *IL17F *(Hs00369400_m1), *IFNG *(Hs00174143_m1), *IL22 *(Hs01574154_m1), *IL23A *(Hs00372324_m1), *IL23R *(Hs00332759_m1), *FOXP3 *(Hs00203958_m1), *IL6 *(Hs00985639_m1), *IDO1 *(Hs00984148_m1), *TBX21 *(Hs00203436_m1) and *GAPDH *(Hs99999905_m1) gene expression. All samples were assessed in triplicate. Samples were considered negative for gene expression when threshold cycle (Ct) values were <40. Positive, test sample Ct values were extrapolated to standard curves obtained from human tonsil or lung standards, to calculate the mean amount of gene-specific RNA in each sample. Results are expressed as the mean ± SEM ng RNA for each gene of interest, relative to the expression of glyceraldehede-3-phosphate dehydrogenase (GAPDH) RNA.

### Double immunofluorescence

Seven-μm sections of frozen synovial tissues were cut and fixed in acetone for 10 minutess at 4ºC. After blocking with 5% goat immunoglobulins (IgGs; Sigma, Saint Louis, Missouri, USA) for 30 minutes at room temperature, the sections were incubated separately with cell-specific mouse monoclonal anti-CD3 (T cells; clone UCHT1), anti-CD20 (B cells; clone L26; DAKO, Glostrup, Denmark), anti-CD14 (monocytes/macrophages; clone FMC-17), anti-DCs (clones CMRF44 [32] and CMRF56 [33,34]), anti-CD303(BDCA-2) (pDCs; clone AC144; Miltenyi Biotec), anti-CD1 (imDC; clone Na134), or anti-prolyl 4-hydroxylase (fibroblast; clone 5B5; Abcam, Cambridge, UK) overnight at 4ºC, followed by incubation with AlexaFluor568-conjugated goat anti-mouse antibody (diluted 1:1500; Invitrogen) for 1.5 hr at 4ºC. The sections were then blocked with 5% mouse IgGs for 30 minutes at room temperature following by incubation with biotinylated mAbs either against AHR (clone RPT9; Abcam) or CYP1A1 (clone b-2; Santa Cruz Biotechnology, Santa Cruz, California, USA) overnight, at 4ºC with subsequent incubation with AlexaFluor488-conjugated streptavidin (10μg/ml; Invitrogen) for 30 minutes, at RT. Cell nuclei were identified by counterstaining with Hoechst 33342 (2.5 μg/ml). Negative controls included the omission of primary antibodies and substitution of primary antibodies with mouse-named IgGs (DAKO). Co-localization of fluorescent staining was assessed by epifluorescence microscopy using a Zeiss microscope fitted with SpotRT digital camera and imaging software (Diagnostic Instruments, Sterling Heights, Michigan, USA). All samples were assessed by a single observer (MK) in a blinded fashion and verified by a second observer (PAH).

### Statistical analysis

Differences in gene-expression levels were analyzed by the Mann-Whitney *U*-test. Where appropriate data was log transformed to stabilise the variance. The one-way analysis of variance (ANOVA) multirange post hoc Tukey's test was used when multiple comparisons were analyzed. Fisher's exact probability test was used to measure association. Correlation coefficients were determined by rank correlation using a nonparametric Spearman test. Analyses were done using Prism for Windows v4.03 (GraphPad Software Inc, San Diego, California, USA) or STATA11 statistical software. A probability level of 5% was considered significant for all statistical analysis.

## Results

### Smoking contributes to AHR activation in inflamed synovial tissue

**Figure 1:**
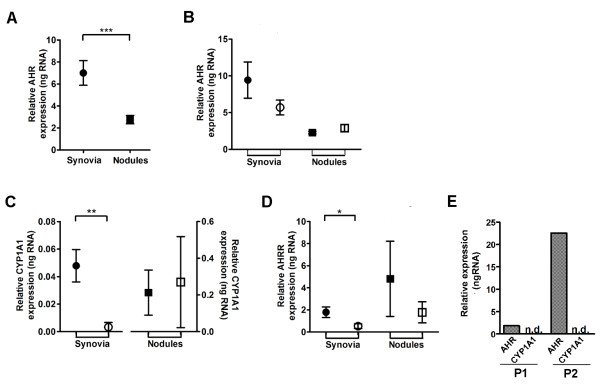
**Aryl hydrocarbon receptor (*AHR*) expression and activation in inflamed rheumatoid tissues**. (**A**) Overall *AHR *expression in synovial tissues (closed circles, n = 20) and nodule tissues (closed squares, n = 18). (**B**) *AHR*, (**C**) *CYP1A1 *and (**D**) AHR receptor (*AHRR*) expression in synovial tissues from rheumatoid arthritis (RA) patients who smoked (closed circles, n = 7) and non-smokers (open circles, n = 13), and nodule tissues of RA patients who smoked (closed squares, n = 4) and non-smokers (open squares, n = 14). (**E**) *AHR *and *CYP1A1 *expression in synovia from two separate patients with osteoarthritis (OA), labelled P1 and P2. n.d., not detected. Data are mean ± standard error of the mean (SEM). All mRNA levels are expressed relative to glyceraldehede-3-phosphate dehydrogenase (GAPDH). ***P < 0.001; **P < 0.01; *P < 0.05, Mann-Whitney *U*-test.

*AHR *expression was present in both synovial tissues and nodules. Expression was higher in synovia (7.00 ± 1.12 ng RNA, n = 20) compared to nodules (2.76 ± 0.37 ng RNA, n = 18, *P *< 0.0006) (Figure 1A), but these levels of *AHR *expression in inflamed rheumatoid tissues were not affected by smoking (Figure 1B).

Expression of the prototypic AHR responsive gene, *CYP1A1 *was used to establish the activation status of AHR in synovial and nodule tissues. CYP1A1 transcript was detected in 7/20 synovial membranes (35% CYP1A1^+^) and in 7/18 nodules (39% CYP1A1^+^). We considered whether smoking was a factor influencing the activation of AHR, reflected by the *CYP1A1 *expression. We found six of the 7 CYP1A1^+ ^synovia were from smokers, whereas 12/13 CYP1A1^- ^synovia were from patients who were non-smokers. The association between patients who smoke and the activation of AHR was significant in inflamed synovium (Fisher's exact test, *P *= 0.005) (Table 2) but was not evident in the separate cohort of 18 patients who provided nodule tissues (Table 2).

**Table 2:** Patient smoking status and tissue aryl hydrocarbon receptor (AHR) activation

Patient Tissue contribution		Patient smoking status*		
			
		Smoker	Non-Smoker	*P*-value^‡^
**Synovia **	CYP1A1^+^	6**^†^**	1	
	CYP1A1^-^	1	12**^†^**	
				0.005
**Nodules**	CYP1A1^+^	2	4	
	CYP1A1^-^	2	10	
				0.57

*Patient smoking status when tissue sample was taken; ^†^Patients contributing paired samples were considered once for statistical analysis; ^‡^*P*-value obtained by Fisher's exact probability two-tailed test

Levels of *CYP1A1 *expression were also significantly higher in synovial tissues from RA patients who smoked compared to non-smokers (0.048 ± 0.011 vs. 0.003 ± 0.003 ng RNA respectively, *P *= 0.004). There was no such difference in nodule tissues (0.21 ± 0.12 vs. 0.27 ± 0.25 ng RNA, Figure 1C). Similarly, expression of the *AHRR *gene, which also depends on AHR activation and consequent transcription activity [35], was significantly higher in synovial tissues from RA patients who smoked when compared to non-smokers (1.79 ± 0.48 vs. 0.53 ± 0.21 ng RNA, *P *= 0.036); this was not the case in nodule tissues (4.82 ± 3.41 vs. 1.78 ± 0.95 ng RNA respectively, Figure 1D). There was a statistically significant positive correlation between *CYP1A1 *and *AHRR *expression in synovial tissues (Spearman r = 0.71, *P *= 0.0005) that also occurred in nodules (Spearman r = 0.62, *P *= 0.0062, data not shown).

Variable expression of *AHR *was also observed in synovia from separate patients with OA. Expression levels overlapped with the range established for rheumatoid synovia (Figure 1E). Irrespective of the extent of *AHR *expression, *CYP1A1 *expression was not detected in OA synovia.

Possible long-term effect of smoking on *AHR *expression and activation within synovial tissue was considered. Three patients providing four synovial samples were ex-smokers, having ceased smoking ≥ 8 years prior. Levels of synovial *AHR *expression were equivalent between patients who were smokers (n = 7), ex-smokers (n = 4) and those who had never smoked (n = 9), consistent with a lack of effect from smoking on *AHR *expression (Additional file 1). *CYP1A1 *expression was not detectable in ex-smokers, suggesting no long-term effect from smoking on AHR activation (Additional file 1).

### Inflammatory genes that reflect the downstream influence of AHR-activation

**Figure 2:**
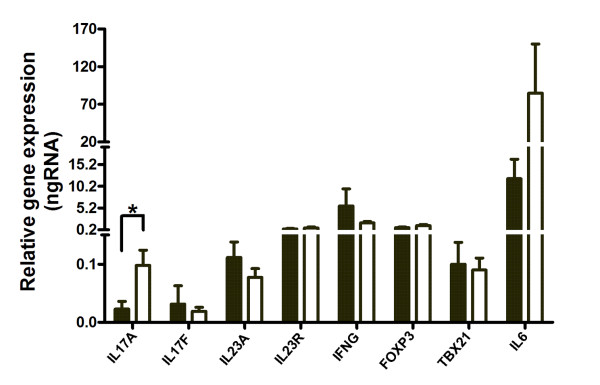
**The effect of smoking on immune-inflammatory gene expression levels in rheumatoid arthritis (RA) synovia**. The figure shows mean gene expression levels ± standard error of the mean (SEM) in synovia obtained from patients with RA who smoked (solid bars) compared to non-smokers (open bars). All mRNA levels are expressed relative to glyceraldehede-3-phosphate dehydrogenase (GAPDH). *P < 0.05, Mann Whitney *U*-test.

We further considered the implications of smoking for the expression of a number of immuno-inflammatory genes implicated in RA. Experimentally, Th17 cells are a direct cellular target of AHR agonists [26]. Their signature cytokine, IL-17A has been implicated in the pathogenesis of RA [36]. As such, Th17 cells provide a possible link between smoking, AHR activation and the exacerbation of synovial inflammation in RA. However, we found significantly less *IL17A *gene expression in synovial tissue from smokers when compared to non-smokers (Figure 2) and a negative association between *IL17A *and *CYP1A1 *gene expression (Spearman r = -0.51, *P *= 0.022).

Amongst other Th17 cell cytokines, expression of the *IL17F *gene was not affected by smoking but was restricted to CYP1A1^- ^synovia, whereas *IL22 *gene expression was not detected in any synovia (data not shown). We also considered the potential for smoking to impact upstream of *IL17A *but found no evidence of an impact on gene expression of the critical cytokine *IL23*, nor of the IL23 receptor (*IL23R*). Similarly, smoking had no effect on Th1-cell mediated inflammation via interferon-γ (*IFNG*), T-bet (*TBX21*) or *FOXP3 *gene expression in synovia (Figure 2).

### Human DCs are implicated in the response of inflamed synovial tissue to smoking

**Figure 3:**
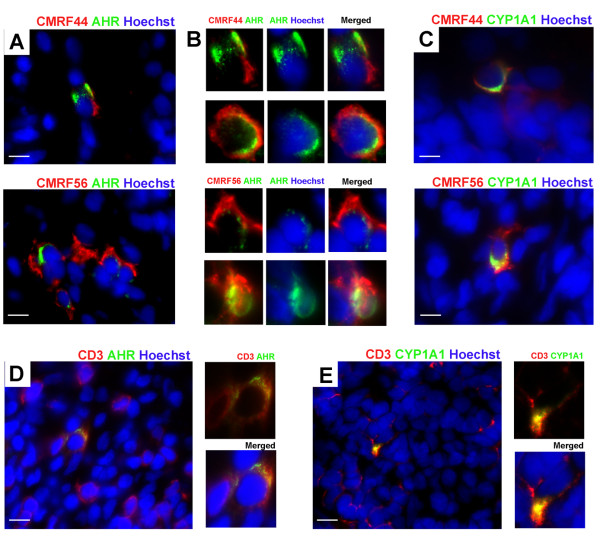
**Human dendritic cells (DCs) are the principal cells implicated in response to smoking**. Double immunofluorescence staining of synovial tissue for (**A**) aryl hydrocarbon receptor **(**AHR) protein (green) in CMRF44^+ ^(red, top panels) or CMRF56^+ ^DCs (red, bottom panels). (**B**) Enlarged images show cytoplasmic and nuclear localization of AHR (green) staining in CMRF44^+ ^(red, top panels) or CMRF56^+ ^DCs (red, bottom panels) as indicated. (**C**) CYP1A1 protein (green) in CMRF44^+ ^(red, top panels) or CMRF56^+ ^DCs (red, bottom panels). (**D**) AHR and (**E**), CYP1A1 protein (green) in CD3^+ ^T cells (red). In all images, nuclei are counterstained with Hoechst (blue). Scale bar, 20 μm.

Next we sought to identify the cell type(s) expressing AHR and showing AHR activation in inflamed synovial tissues. Using double immunofluorescence staining, CYP1A1 protein was observed only in CYP1A1^+ ^synovia from RA patients who were smokers. In these synovia, both AHR and CYP1A1 proteins were produced by early differentiating CMRF44^+ ^and CMRF56^+ ^DCs (Figure 3A, B, C). These AHR^+^/CYP1A1^+ ^DCs were mainly observed in close proximity to T and B cells (data not shown). Staining for AHR was cytoplasmic and in some DCs more obviously perinuclear, with small amounts of AHR protein localized within the nucleus, consistent with AHR activation (Figure 3B). Amongst the other cell types examined, rare (much less than 1%) CD3^+ ^T cells produced AHR and CYP1A1 proteins (Figure 3D, E). Although most CD303^+ ^plasmacytoid DCs (pDCs) produced AHR protein, only occasional CD20^+ ^B cells were AHR^+ ^and neither pDCs nor B cells produced detectable CYP1A1 protein. In synovium, immature CD1^+ ^DCs, CD14^+ ^monocyte/macrophages and fibroblasts expressing prolyl-4-hydroxylase did not express either AHR or CYP1A1 protein (data not shown).

AHR or CYP1A1 protein was not detected in nodule lesions, regardless of smoking status (data not shown).

### Human mo-DCs are highly sensitive to AHR stimulation with BaP

**Figure 4:**
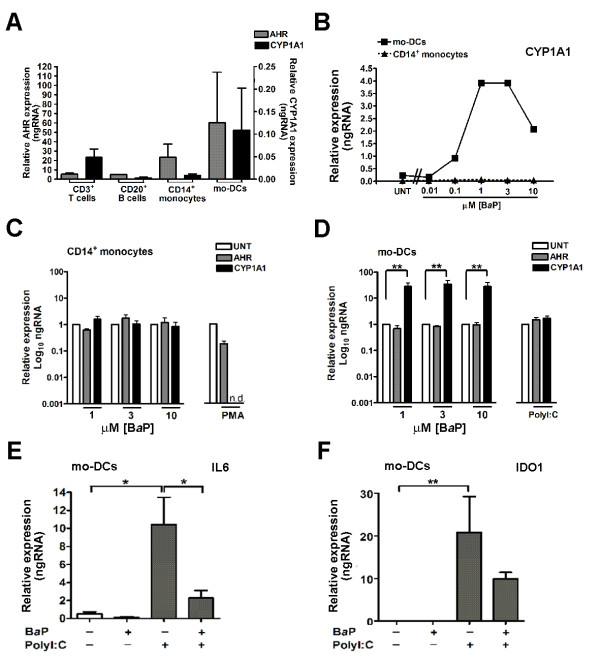
**Increased sensitivity of monocyte-derived dendritic cells (mo-DCs) to benzo(*a*)pyrene (B*a*P) stimulation**. (**A**) The expression of aryl hydrocarbon receptor **(***AHR*) and *CYP1A1 *in human peripheral blood (PB) CD3^+ ^T cells, CD20^+ ^B cells, CD14^+ ^monocytes and mo-DCs. Gene expression was analyzed using qRT-PCR. All mRNA levels are expressed relative to glyceraldehede-3-phosphate dehydrogenase (GAPDH). Data are the mean ± standard error of the mean (SEM) (n = 2). (**B**) Comparison of the dose-dependent response of CD14*^+ ^*monocytes and mo-DCs to (0.01-10μM) B*a*P stimulation (24 hr). Data are representative of three experiments with cells purified or derived from the same PB sample. Comparison of *AHR *and *CYP1A1 *gene expression in (**C**) CD14^+ ^monocytes and (**D**) mo-DCs when stimulated with varied concentrations (1 - 10μM) of B*a*P (24 hr), 10μM phorbol 12-myristate 13 acetate (PMA) (72 hr; monocytes), or polyinosinic:polycytidylic acid (PolyI:C) (24 hr; mo-DCs) treatment. Data are the mean ± SEM (n = 3) relative to AHR and CYP1A1 mRNA levels found in untreated cells (UNT), arbitrarily set at a value of 1 with cells purified or derived from the same PB sample. (**E**) Expression of *IL6 *and (**F**) *IDO1 *in response to 3μM B*a*P (24 hr) or PolyI:C (24 hr) alone, or in combination (as indicated). n.d., not detected. Data are the mean SEM from 3 experiments. *P < 0.05, **P < 0.01, Tukey multiple comparison test.

Finally we questioned the type(s) of cells that respond to AHR ligands with evidence of AHR activation. We found comparable *AHR *gene expression in T and B cells and monocytes from normal human PB cells and in mo-DCs (Figure 4A). Monocytes showed no significant change in *AHR *or *CYP1A1 *expression when exposed to increasing concentrations of the AHR agonist, B*a*P (Figure 4B,C). In contrast, mo-DCs were extremely sensitive to B*a*P. The inducing effect of B*a*P upon *CYP1A1 *expression in mo-DCs was evident at 0.1 μM and maximal at B*a*P concentrations of 1-10μM (Figure 4B, D), eliciting an average 30-fold increase in *CYP1A1 *expression (Figure 4D). When compared to untreated cells we observed no effect on *AHR *or *CYP1A1 *expression in PMA-treated monocytes (Figure 4C) or when mo-DC maturation was induced by PolyI:C (Figure 4D).

We sought evidence for a possible mechanism linking smoking, DC function and the effect on *IL17A *expression. Significant *IL6 *gene expression was a feature of mo-DCs induced to mature via PolyI:C stimulation (Figure 4E). This expression was inhibited by the AHR agonist, B*a*P (Figure 4E). Significant *IDO1 *expression was also a feature of PolyI:C-induced mo-DC maturation. However this expression was not affected by B*a*P (Figure 4F).

## Discussion

It is now well established that interactions between smoking and the SE increase the risk of developing RA [37] through effects mediated in the early pre-clinical stage of RA [38,39]. There are also indications that in established RA smoking is associated with more active disease and worse outcomes [20,21]. We hypothesized that in established RA, products within cigarette smoke, such as PAHs, may act upon the AHR, resulting in activation and downstream pro-inflammatory effects. *In vitro *work has suggested that this might include activation of Th17 cells, a pathway established as important to promotion of inflammation and joint damage in RA. We therefore sought evidence for *AHR *expression in RA lesions, the activation of AHR, and cells that showed evidence of AHR-mediated activation.

In advanced, erosive RA, we demonstrated *AHR *expression in both synovial and subcutaneous nodule tissues. There was significantly more *AHR *gene expression in synovial membrane but this expression was not affected by smoking. The nodule and synovium are different inflammatory lesions with respect to the composition of the cellular infiltrate. Subcutaneous nodules are granulomas, dominated by monocyte/macrophages but our data suggest that the difference in *AHR *gene expression is not entirely due to this difference in inflammatory cell type. Limited analysis of synovia from patients with OA also indicated *AHR *expression suggesting that inflammation, particularly associated with RA, is not entirely responsible for upregulated AHR expression.

Only in synovial tissue did we find evidence that smoking causes significant AHR activation, reported by increased expression of both the *CYP1A1 *and *AHRR *genes. Amongst patients who smoked, the majority showed this pattern of AHR-mediated activation. While was little evidence of AHR activation in synovium from non-smokers, there was also no indication that *AHR *expression or activation in synovia from ex-smokers was different from patients who had never been smokers. Any evidence of AHR activation within synovial tissue was gone 8 to 33 years after ex-smokers ceased smoking, suggesting no lasting impact of smoking on AHR-mediated mechanisms. Such a finding is at odds with the long-term effect of smoking on the risk of developing RA [8] and indicates that the activation of AHR by current smoking might be more important to ongoing synovial inflammation and thereby contribute to the severity of established RA. Consistent with this, a recent report also distinguishes an effect from current smoking, manifest as a reduced chance of response to methotrexate and to TNF inhibitor treatment. This compares with past smoking, which has no such influence on treatment [40]. Thus the risk of RA from smoking likely relates to a mechanism(s) independent of continued AHR activation.

The situation in nodules is more complex. In nodule tissues we found generally higher levels of *CYP1A1 *gene expression but were unable to demonstrate CYP1A1 protein. However, the most important observation was that *CYP1A1 *gene expression in nodules was independent of current smoking status or smoking history. This suggests an alternative (possibly endogenous) AHR ligand within these lesions, the nature of which remains to be determined. It suggests that the AHR-mediated response to cigarette smoke may be most pronounced in the inflamed synovium in patients with RA. Whether this is a unique aspect of RA remains to be established. Synovia from OA patients were CYP1A1-negative regardless of *AHR *expression levels. Unfortunately, details of these patients' smoking status were unavailable.

While our data indicate smoking has a molecular impact within inflamed rheumatoid synovium, a critical question is how any AHR-mediated mechanism might influence the inflammatory process. We addressed this question by identifying the cell types within synovium that showed evidence of AHR activation. We found that a subset of CMRF56^+ ^and/or CMRF44^+ ^DCs within inflamed synovia were the predominant cells that expressed both AHR and CYP1A1 protein. There were also very occasional T cells and plasmacytoid DCs and, even more rarely, B cells in these synovia that expressed AHR protein. Amongst these non-DC types, only the T cells showed evidence of AHR activation through CYP1A1 protein expression. *In situ*, these T cells were rare, even in synovia where generalized T cell infiltration was prominent. Synovial monocyte/macrophages were negative for both AHR and CYP1A1 protein. Clearly AHR^+^/CYP1A1^+ ^DCs within the synovium display markers characteristic of early differentiating/activated DCs. The data highlight these cells as a potential focal point for the effects of cigarette smoke components within tissues and subsequent immune/inflammatory outcomes.

We considered further detail of mechanisms potentially utilized by DCs in response to AHR agonists. For this we used B*a*P, an AHR agonist that can be metabolized and is a known component of cigarette smoke [41]. When compared to paired PB CD14^+ ^monocytes, mo-DCs responded to lower concentrations of B*a*P *in vitro*, with AHR activation that culminated in significantly greater *CYP1A1 *expression at higher B*a*P concentrations. Thus, DCs are more sensitive to B*a*P, an observation that is consistent with these being the most commonly observed cells showing evidence of AHR activation in the inflamed synovial tissue. Similarly, the insensitivity of monocytes to B*a*P is consistent with the lower *AHR *gene expression and activation, and lack of detectable protein observed in monocyte/macrophage-dominated nodules. There are known immune/inflammatory consequences of DCs responding in such a sensitive manner to AHR agonists. In murine systems AHR activation can directly affect the differentiation and innate immune functions of inflammatory DCs without affecting their ability to interact with T cells [42,43]. Overall the studies in mice suggest AHR-mediated mechanisms influence the regulatory activities of DCs with consequences that skew naïve T cell differentiation towards Treg cells and away from Th17 cells. Such a response would be expected to limit immune/inflammatory activity [44,45]. We observed reduced expression of *IL17A *in synovia from RA patients who smoked, a negative correlation between *IL17A *expression and AHR activation in synovia, and expression of *IL17F *that was limited to CYP1A1^- ^synovia. All are consistent with an effect of smoking on Th17 cell systems that involves AHR activation within intermediary DCs, rather than a direct effect on actual Th17 cells. As discussed, there was limited evidence of synovial T cells responding directly to smoking. There was no change in the presence of regulatory T cells (measured as *FOXP3 *expression), Th1 cells or Th1-like, ex-Th17 cells (*TBX21*/T-bet and *IFNG *expression) producing interferon-γ [46] between synovia from smokers and non-smokers. While expression of the *IL6 *and *IL23 *genes was not significantly different between these synovia, the data were indicative of some effect on *IL6 *from current smoking. This possibility was strengthened by *in *vitro data showing that B*a*P exposure inhibits *IL6 *expression by PolyI:C-stimulated mo-DCs. In this manner a critical regulatory signal, operative at the DC-T cell interface, could be affected by AHR activation in DCs, ultimately influencing DC control of T cells. Our data are consistent with this control negatively impacting on Th17 cells. We cannot exclude direct effects on T cells suggested by the rare AHR^+ ^T cells observed in synovia.

Such effects on potential or relevant pathogenic mechanisms contrast with epidemiological evidence showing increased risk of disease and worse inflammation in patients with RA who smoke. Interactions between smoking and the presence of SE^+ ^*HLADRB1 *alleles offer some explanation for this anomaly. All patients in our study were SE^+ ^thereby precluding detailed comparison of SE presence or copy number. Preliminary analysis, including this patient cohort, suggests an opposing effect of smoking and SE copy number on synovial IL17A expression.

In summary we have demonstrated the presence of the AHR receptor within synovial tissue from patients with RA, with the potential to interact with polycyclic aromatic hydrocarbons in cigarette smoke. We have found evidence for activation via the AHR with increased levels of *CYP1A1 *and *AHRR *in synovial tissues from patients who continue to smoke. Based on *in vitro *data, we considered that such AHR activation, mediated by products in cigarette smoke, might activate Th17 cells. In contrast to this we found decreased expression of *IL17*, and that the predominant cell activated was the DC. Indications are that reduced *IL6 *expression by DCs is a possible consequence of cigarette smoke exposure, a factor that subsequently impacts negatively on *IL17A *expression.

## Conclusions

Our data suggests that cigarette smoke-induced changes in DC responses have the potential to be relevant to both the early pre-clinical phase of RA and the later stages of the disease. In the pre-clinical phase it is thought that antigen-presenting cells that express the shared epitope preferentially interact with post-translationally altered citrullinated antigens. Smoking is thought to act through promoting the citrullination of relevant antigens. Our data suggest that smoking might also act by altering DC responses. This effect on DCs could also be relevant to the influence of smoking on inflammation in established RA. Further detail of the effects of smoking on DC has the potential to provide further insights into mechanisms relevant to both the early induction phase of RA and worsening of established RA.

## Abbreviations

ACPA: anticitrullinated peptide antibody; AHR: aryl hydrocarbon receptor; AHRR: AHR repressor; BαP: benzo(*a*)pyrene; Ct: threshold cycle; CYP: cytochrome P450; DMARDs: disease-modifying anti-rheumatic drugs; DMSO: dimethylsulphoxide; GAPDH: glyceraldehede-3-phosphate dehydrogenase; IL: interleukin; Mo-DC: monocyte-derived dendritic cell; OA: osteoarthritis; PAHs: polycyclic aromatic hydrocarbons; PB: peripheral blood; PMA: phorbol 12-myristate 13 acetate; Poly I:C: polyinosinic:polycytidylic acid; RA: rheumatoid arthritis; RF: rheumatoid factor; rh: recombinant human; RT-PCR: real-time polymerase chain reaction; GMCSF: granulocyte, macrophage colony stimulating factor; SE: HLADRB1 shared eptiope; SEM: standard error of the mean; Th: T-helper; TNF: tumor necrosis factorα; XRE: xenobiotic response element.

## Competing interests

The authors declare that they have no competing interests.

## Authors' contributions

MK carried out the majority of the experimental work and statistical analysis, and drafted the manuscript. JH and LS coordinated patient recruitment, clinical assessment and sample recovery, participated in the design of the study, and helped draft the manuscript. PH conceived of the study, participated in its design and coordination, and helped draft the manuscript. All authors read and approved the final manuscript.

## Supplementary Material

Additional file 1 **Long-term effect of smoking on aryl hydrocarbon receptor (*AHR*) expression and activation in synovial tissues**. Shown is any effect from current smoking or former smoking on *AHR *expression and *CYP1A1 *gene expression in joint synovial tissue from RA patients. Comparisons are made with data from non-smokers. There is no significant difference in synovial AHR expression between RA patients who are current smokers, ex-smokers or non-smokers. The expression of *CYP1A1*, reflecting AHR activation, is significantly different only in rheumatoid arthritis (RA) patients who continue to smoke. Click here for file
