# Supplementary material for: Dendritic cells provide a potential link between smoking and inflammation in rheumatoid arthritis
Source: Arthritis Res Ther. 2012 Oct 4;14(5):R208. doi: 10.1186/ar4046 (PMC3580520; doi:10.1186/ar4046)
Supplement: Additional file 1 — Long-term effect of smoking on aryl hydrocarbon receptor (AHR) expression and activation in synovial tissues. Shown is any effect from current smoking or former smoking on AHR expression and CYP1A1 gene expression in joint synovial tissue from RA patients. Comparisons are made with data from non-smokers. There is no significant difference in synovial AHR expression between RA patients who are current smokers, ex-smokers or non-smokers. The expression of CYP1A1, reflecting AHR activation, is significantly different only in rheumatoid arthritis (RA) patients who continue to smoke. [file ar4046-S1.PDF]

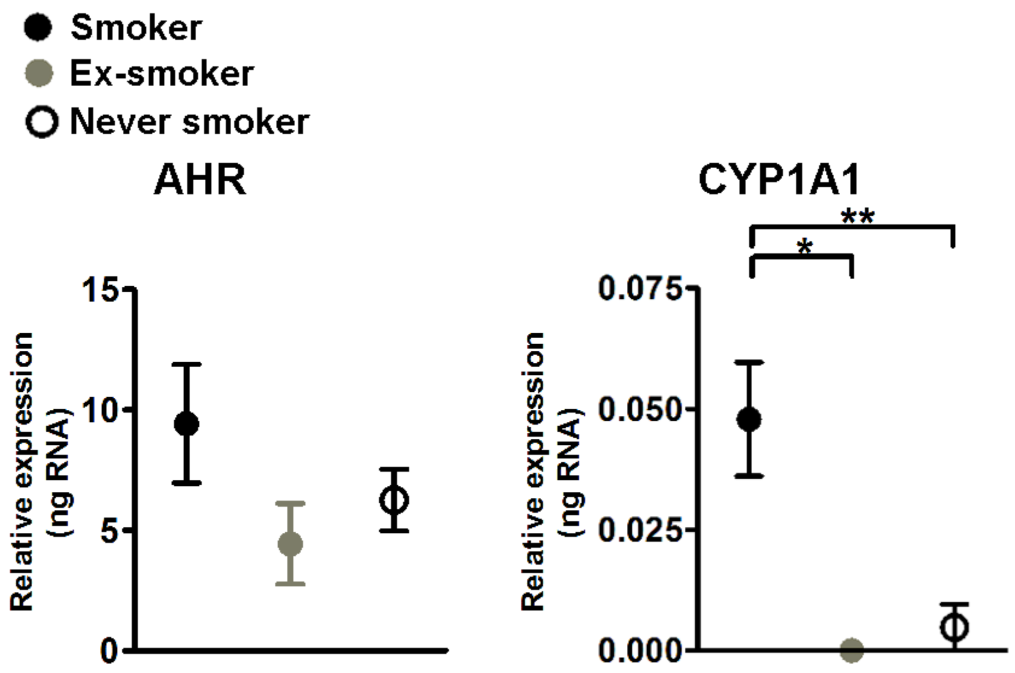

Additional file 1, Figure S1. Long-term effect of smoking on AHR expression and activation in synovial tissues. Figure shows AHR and CYP1A1 expression in synovial tissues from RA patients who are smokers (n = 7), ex-smokers (n = 4) or never smokers (n = 9). Data are mean ± s.e.m. All mRNA levels are expressed relative to GAPDH. $^{**}P < 0.001$; $^{*}P < 0.05$ by Mann-Whitney U test.
